# Health Extension Workers' and Mothers' Attitudes to Maternal Health Service Utilization and Acceptance in Adwa Woreda, Tigray Region, Ethiopia

**DOI:** 10.1371/journal.pone.0150747

**Published:** 2016-03-10

**Authors:** Ruth Jackson, Fisaha Haile Tesfay, Hagos Godefay, Tesfay Gebregzabher Gebrehiwot

**Affiliations:** 1 Alfred Deakin Institute for Citizenship and Globalisation, Deakin University, Geelong, Victoria, Australia; 2 College of Health Sciences, Department of Public Health, Mekelle University, Mekelle, Tigray Region, Ethiopia; 3 Tigray Regional Health Bureau, Mekelle, Tigray Region, Ethiopia; Banaras Hindu University, INDIA

## Abstract

**Background:**

The maternal health system in Ethiopia links health posts in rural communities *(kebeles)* with district *(woreda)* health centres, and health centres with primary hospitals. At each health post two Health Extension Workers (HEWs) assist women with birth preparedness, complication readiness, and mobilize communities to facilitate timely referral to mid-level service providers. This study explored HEWs’ and mother’s attitudes to maternal health services in Adwa Woreda, Tigray Region.

**Methods:**

In this qualitative study, we trained 16 HEWs to interview 45 women to gain a better understanding of the social context of maternal health related behaviours. Themes included barriers to health services; women’s social status and mobility; and women’s perceptions of skilled birth attendant’s care. All data were analyzed thematically.

**Findings:**

There have been substantial efforts to improve maternal health and reduce maternal mortality in Adwa Woreda. Women identified barriers to healthcare including distance and lack of transportation due to geographical factors; the absence of many husbands due to off-*woreda* farming; traditional factors such as *zwar* (some pregnant women are afraid of meeting other pregnant women), and discouragement from mothers and mothers-in-law who delivered their children at home. Some women experienced disrespectful care at the hospital. Facilitators to skilled birth attendance included: identification of pregnant women through Women’s Development Groups (WDGs), and referral by ambulance to health facilities either before a woman’s Expected Due Date (EDD) or if labour started at home.

**Conclusion:**

With the support of WDGs, HEWs have increased the rate of skilled birth attendance by calling ambulances to transfer women to health centres either before their EDD or when labour starts at home. These findings add to the growing body of evidence that health workers at the community level can work with women’s groups to improve maternal health, thus reducing the need for emergency obstetric care in low-income countries.

## Introduction

Reducing maternal mortality by three quarters by 2015 was endorsed as a major international development goal at the Millennium Summit in 2000 [1]. In 2013, sub-Saharan Africa accounted for 62 percent of global maternal deaths: Ethiopia’s Maternal Mortality Ratio (MMR) was estimated to be 420 maternal deaths per 100,000 live births with a lifetime risk of maternal death of one in 52 [2]. Other estimates include the 2011 Ethiopian Demographic and Health Survey (EDHS) where the MMR was estimated to be 676 maternal deaths per 100,000 live births [3]—almost the same level as the 2005 EDHS MMR level of 673 per 100,000 live births [4]. The target is to reduce the MMR to 267 deaths per 100,000 live births by the end of 2015 [5, 6].

While there has been international consensus about prioritizing maternal mortality reduction and improving maternal health, the strategies on how to go about it have not always been agreed on at a contextual level [7, 8]. Preventing maternal death is not as simple as it is for other conditions such as vaccine-preventable diseases, but most agree that functioning health systems are crucial. There have been disagreements about the complexity of the interventions; the evidence base for these interventions and their cost [9] particularly when facility based interventions do not reach the poorest households [10]. There has also been tension between those advocating facility-based care and Emergency Obstetric and Newborn Care (EmONC) that focus on maternal survival, while those advocating newborn and child survival place more emphasis on community-based strategies [11, 12]. In brief, there are two approaches to health system strengthening in low-income countries: the first, developed by the WHO is to build more health centres staffed by physicians, nurses and midwives, while the second approach developed by the World Bank, UNICEF, UNFPA and the Partnership for MNCH is to develop more health posts with basic training at the community level [13].

Ethiopia’s approach has been to combine both health system approaches by building more health facilities and staffing them with skilled birth attendants, and increasing the number of basic trained health workers—Health Extension Workers (HEWs)—at the community level. The Health Sector Development Programme (HSDP), a 20-year community-oriented health development strategy implemented through four consecutive five-year programmes, aims to decentralize the health care system; develop preventive and curative components of health care; and to ensure accessibility of health care to all segments of the population [5].

HSDP IV is a three-tier system characterized by a first-level district (*woreda)* health system comprising a primary hospital (with population coverage of 60,000–100,000 people), health centres (1/15,000–25,000 population) and five satellite health posts (1/3,000–5,000 population) that are connected to each other by a referral system. The second level tier is a general hospital with population coverage of 1–1.5 million people, and the third a specialized hospital that covers population of 3.5–5 million. According to the Federal Ministry of Health (FMOH) in 2013/14 there were 16,251 health posts, 3,335 health centres and 156 public hospitals, an increase from 14,192 health posts, 2,142 health centres and 126 hospitals (all types) in 2009/10 [5, 14]. The main challenges for HSDP IV is to ensure that the functionality of the health infrastructure and the quality of health services keep pace with the expansion in health facilities [15].

The *Road Map for Accelerating the Reduction of Maternal and Newborn Morbidity and Mortality in Ethiopia* aims to increase skilled attendance during pregnancy, childbirth and the postnatal period; scale up basic and comprehensive EmONC; and, increase access to family planning [6]. The introduction of the Health Development Army (HDA), the network at the community level that focuses on local behaviour change, aims to improve the uptake of Maternal, Newborn and Child Health (MNCH) services. All *kebeles* should now have a women-centred health development teams comprising of up to 30 women and a one-to-five network of women to enhance and consolidate the implementation of the Health Extension Program (HEP). Activities include monthly meetings with pregnant women to discuss birth preparedness and complication readiness; facilitation of the participation of health workers from primary health care units; the importance of Antenatal Care (ANC) and skilled attendance at delivery; family planning; the negative health and social consequences of harmful traditional practices; and, proper nutrition and micronutrients.

The *Road Map* also stresses the involvement of other community members to promote institutional delivery, to mobilize resources for emergency blood donation and transport, and the empowerment of women, men, families and communities to take responsibility for developing and implementing MNCH. HDA activities include timely notification to HEWs of women in labour and deliveries, capacity building of the HDA to recognize and refer maternal/newborn danger signs, and training HEWs in clean and safe delivery, newborn care and postnatal care.

Although HEWs were initially trained in clean and safe delivery, they are now expected to refer all women to health centres either before their Expected Due Date (EDD) or if labour starts at home. While studies show that the HEP has been well received and that the use of family planning and ANC by women has increased, the impact on the program on other maternal health indicators such as skilled assistance at birth has been limited [16–25]. For example, one study showed that less than a quarter of pregnant women were well prepared for delivery and emergency obstetric care, less than half (44.7%) had attended ANC and most women (87.9%) intended to give birth at home [26].

In Tigray Region in northern Ethiopia, the Tigray Regional Health Bureau (TRHB) has made considerable efforts to improve MNCH services. However, there are still strong cultural and traditional factors which are considered common barriers to skilled birth attendance including: considering birth as a natural event; absence of women’s decision making capacity during delivery; elder influence; and relying on God and Saint Mary *(Mariam)* for a safe home delivery. Health system factors such as the unavailability of transport, low competency of health workers, poor referral linkage and negative interaction of health service providers are other challenges [27, 28]. Therefore, this research explores HEWs and mothers’ attitudes to maternal health service utilization and acceptance using a peer informant approach.

## Methods

### Setting

The research took place in Adwa Woreda, Central Zone, Tigray Region in northern Ethiopia, 1015 kms from the capital Addis Ababa. Tigray is a land of highland plains, mid-land plateaus, valley bottoms and vast escarpments with mountains ranging from 500 metres in the west to the Tsibet Mountains 3,935 meters above sea level in the south. Adwa is one of the 34 rural *woredas* located in Central Zone with an estimated population of 107,953 [29].

The research sites were selected in consultation with the TRHB [author HG] and subsequently the Adwa Health Office. All 18 *kebeles* were invited to send one HEW to attend the workshop in Adwa town but only 16 HEWs attended. At the time of the research one ambulance linked the 18 health posts with seven health centres (at the *woreda* level), and the health centres with Adwa Hospital through a referral system so that women could be transferred if there was an obstetric emergency. A second ambulance was under repair.

The HDA evolved differently in Tigray Region compared to other parts of Ethiopia due to the political leadership and commitment by all levels of health professionals and the community. The community is represented by Women’s Development Groups (WDGs) for maternal health issues. WDGs are seen as the cornerstone of community ownership, making invaluable contributions within the health sector as well as in other development sectors. They are ‘conceptualized as a way to create demand for health, wellness, and improved access to health care services through organized voluntary groups of women’ [30].

According to the FMOH, the percentage of deliveries assisted by skilled health personnel in Ethiopia has shown a steep increase from 23.1% in 2012/13 to 40.9% in 2013/14 [14]. However, there is still no consistent figure of skilled birth attendance—and the 2014 Mini EDHS report indicated that for the five years prior to the survey only 15% of women gave birth in a health facility [31]. According to the TRHB, just over half (54%) of women in Tigray Region were assisted by skilled health personnel [30]. Statistics provided by the Adwa Health Office for the first quarter of 2013/14 show that 29% of women gave birth in a health centre, 4% in a health post and 2% at home. For the first three months of 2014/15 statistics showed that of the 894 women who planned to give birth, 238 received skilled attendance at the health centre and six women were assisted by HEWs through safe and clean delivery at home. An additional 174 women were referred either from home to a health centre, or from health centre to hospital. There was one maternal death in Adwa Hospital [32].

### Study design

The methods for this project were adapted from Key Informant Monitoring and Participatory Ethnographic Evaluation and Research [33–35]. HEWs were selected because they are generally women who come from the same *kebele* as the women they were interviewing and because they hold a trusted position as health workers in the *kebele*. In particular, women appreciate the concern of HEWs whose important role on maternal health services influences women regarding ANC and institutional delivery utilization [27]. We expected that HEWs would encourage a certain level of openness from women they knew that we may not have gained ourselves. In this research, HEWs were both research assistants and informants. On the one hand, HEWs with better understanding to traditional and cultural values of the community can be considered as informants of the study, and on the other hand, they are also considered as gatekeepers to health care and mediators between the traditional and medical model of care which was a good justification for recruiting them as research assistants.

HEWs attended a two-day workshop in Adwa town where they were trained how to conduct research ethically; interview techniques; how to develop their own research questions and design an interview schedule; how to recruit participants; and how to identify key issues and incorporate lessons learnt into their practices. We practised asking open-ended questions, probing, and asking for stories; third-person interviewing; how to record findings and identify key phrases and/or events the interviewees gave most importance to. We provided each HEW with a form to record women’s responses with questions listed under the three broad themes agreed to during the workshop in detail: barriers to existing health services and perceptions of quality of care; women’s social status and mobility; and attitudes to childbirth including increasing utilization and acceptance of skilled birth attendants and early referral. Minor modifications were made to the data collection forms during the workshop based on comments by the HEWs and the researchers. This ensured the form was standardized by creating common understanding about what information was to be collected. During visits to the HEWs the research team was able to clarify any questions to further ensure the standardization of the interviews.

After the workshop, HEWs returned to their *kebeles* where they identified and obtained informed consent from three women. HEWs were asked to select women to interview who had had a range of experiences giving birth in the previous two years: at home or in a health facility, and women who had had one or two children or more than two children. As HEWs live and work in the community, it was technically possible for them to identify women who had had positive or negatives experiences. The aim of the study was not generalizability based on a representative sample but to identify and understand the barriers of maternal health care utilization as experienced by women with divergent birth experiences. Women were interviewed by the HEW in the following week. All of the HEW interviews with women were one on one. We conducted semi-structured one on one interviews with HEWs and other health workers to assess their perceptions about the utilization of maternal health services.

## Data collection

Data were collected in November 2014. Authors RJ and FH conducted the workshop and interviews. Inclusion in the research was by invitation from the Adwa Health Office to one HEW from each health post—generally the HEW with the most experience. 16 out of 18 HEWs agreed to participate in the research and interviews. None of the HEWs or women who were interviewed by the HEWs refused to take part in the research. There were no prior relationships with any participants. The workshop and all interviews were conducted in English (RJ) with translations into Tigrinya (FH). RJ is female and FH is male. Data collected by HEWs was translated from Tigrinya into English by author FH. Field notes rather than audio recording was used by HEWs and the research team.

### Ethics

Ethical approval was obtained from the Deakin University Human Ethics Committee (2013–055) and the Ethiopian Federal Ministry of Science and Technology National Research Ethics Review Committee (Phase 27, No 189). All participants were informed about the purpose of the study, the interview procedures, and their right to refuse and withdraw at any time in a language they could understand (Amharic or Tigrinya). Voluntary informed consent was gained from all participants either orally or in writing. HEWs and other health workers signed written consent forms. If the HEWs interviewed women who were unable to read or write, they were asked by the HEW to provide oral consent and then the HEW signed the consent form on behalf of the woman. No women’s names were reported on data collection sheets as HEWs were asked not to report the names of the women they interviewed. The reason for this is that HEWs asked women to talk about, or tell stories about ‘other people like them’ rather than to talk about themselves or to identify people [33–35]. Consent was approved based on the knowledge that many women in rural Ethiopia are not able to give written consent, that the study was not likely to be harmful and that all women interviewed were over the age of 18 and had given birth within one to two years before the study. To ensure confidentiality, HEWs were randomly assigned a number (HEW1, HEW2 and so on).

HEWs were offered travel costs to the workshop and the standard government per diem for attending training. No compensation was offered to the women in the study. HEWs received feedback and the opportunity to share their findings during the final workshop after the data collection period. A detailed report was provided alongside a presentation to the TRHB and the Adwa Woreda health office in August 2015. The collected data has been kept in a locked cabinet (hard copies) or on computers that are password protected (soft copies).

### Data analysis

After data collection, HEWs returned to Adwa for a second workshop to discuss their findings with other HEWs and the research team. This allowed HEWs and the research team to identify key issues emerging from the interviews, and lessons learned. In keeping with the principles recommended by Price *et al*. [33], the purpose was to analyze the data, promote discussion and enable HEWs to contribute to insights and raise awareness about the issues under investigation.

Key themes such as barriers to health services and what makes it easy to access health services were discussed in depth to ensure our interpretation of the data was consistent with what the women told HEWs. However, we were not focused on the frequency of how many women mentioned key barriers for instance, or on collecting other ‘facts’ but were more interested in understanding ‘the different ways in which people talk about and describe the social world they experience around them’ [34]. The women’s narratives served as primary social data with the HEWs serving as the social analyst’s key informants. We benefited from working with HEWs who are the bridge between delivering MNCH programs at the *kebele* level and the women in the *kebeles* they are aiming to reach.

Data were translated into English by FH and analyzed thematically (RJ and FH). Data analysis by the research team involved entering narrative data into Microsoft Word in English, reading and re-reading and identifying key themes. We analyzed the data according to the pre-existing analytical framework developed according to the objective of the research.

## Results

### Characteristics of the women interviewed

16 HEWs interviewed 45 women about maternal health seeking behavior in Adwa Woreda, Tigray Region (one HEW who was around 40 weeks gestation of pregnancy at the time of the workshop did not interview any women). The average age of the women who were interviewed was 32 years. Around two-thirds (31 women) were married and the majority of women (42 out of 45) were Ethiopian Orthodox by religion. Just over half of the women have attended some formal education with the majority (23 women) achieving only primary education. Only 3 of the women had never received a Tetanus Toxoid vaccination, around half the women had had 2 vaccinations while the remaining women had been vaccinated 3 or 4 times. 6 women had received 5 Tetanus Toxoid vaccinations. More than three-quarters of the women (36 out of 45 women) had attended ANC by a midwife at least once during their last pregnancy. 5 of the women had given birth to their last child at home, 3 at the health post, 23 at the health centre and 14 at the primary hospital. 27 women reported that midwives attended the birth of their last child.

### Barriers to skilled birth attendance

Our data revealed that there are still barriers that cause women to deliver at home. These include distance, lack of transportation due to geographical factors and the absence of many husbands who go to Western Tigray to farm for almost six months every year. The reason for the absence of husbands is because the average size of farms in Adwa Woreda is too small to produce adequate cereals for all household members. The absence of many husbands is perceived to influence women’s access to maternal health services such as ANC and institutional delivery, as women do not want to attend if there is no one to look after other children, the cattle and the house. Although a woman’s mother, grandmother, mother-in-law or older relative will stay with her, the husband is still considered the key person who favours seeking health care. Older women are more in favour of traditional cultural practices and traditions. Due to this situation, the decision-making power of women is reduced in the absence of their husbands, especially the cost incurred for transportation and other fees other than the health care costs. Thus, women could be influenced to stay at home and prefer the traditional model of care instead of the medical mode.

HEWs described how many women live far from the road so they may need to be carried by stretcher to the main road. As HEW2 said, “If the woman can walk, we start walking, if not we start carrying her on a stretcher.” And HEW7 explained, “There is a serious problem when men are away. Once there was no one in the sub-*kebele* so they had to call old priests and students to carry a woman on a stretcher.” As one woman told HEW13:

“I walk 5 to 10 kms to collect water each day, sometimes twice a day. My husband is away, to farm in Western Tigray from June to December—our land here is too small. How can I look after the crops and the children? It’s a three-hour walk to the health centre. How can I go for a check-up when there is no one to look after the children?”

Traditional factors such as *zwar* (also known as *kitab)*, means pregnant women are afraid of meeting other pregnant women. *Zwar* refers to the leaves and roots of a special plant that is crushed, put in a small piece of animal skin that is folded and tied, and then tied under the clothes on the arm or around the neck with other things on a necklace and hidden under the dress of the pregnant woman. *Zwar* is considered to prevent problems during pregnancy including miscarriage and to ensure the child grows properly. *Zwar* is perceived as safe for the woman who wears it, but if other pregnant women are not wearing it, they are afraid of those wearing it as it may create problems for their unborn child and themselves. If pregnant women are not wearing *zwar*, it is safe for others to be around them. Although most HEWs stated that *zwar* would not prevent women from coming together to the WDGs or attend ANC clinics with other women, others claimed that some families are more likely to be affected by this than others especially if problems during pregnancy are considered because *zwar* is a traditional belief and the information could be conveyed from mothers to daughters over time.

Until modern health services were introduced in recent years, a Traditional Birth Attendant (TBA) or family member assisted women to give birth at home. Women who had birth at home without any problems may not think it necessary for them to go to the health centre. Or as HEW7 explained:

“The mothers, grandmothers, or mothers-in-law say that they had many children at home so it’s not necessary for a pregnant woman to go to the health facility. Also, many household duties prevent women from going. One woman had a two-year old child and was worrying who would care for her child. The HEW and the *kebele* administrator had to call the husband to come back from Western Tigray to look after the child so she could go to the health facility.”

Some accounts reveal how uncertain and fearful women feel about going to a health centre or hospital. HEW13 described an event which happened a month before the research:

“A woman with hypertension was admitted to Adwa Hospital but she suddenly escaped and went home. The doctor called the health centre staff and subsequently the health centre staff called the HEW but the woman was hiding. They called the police and the *kebele* administrator to search for the woman but couldn’t find her. The husband denied that she was in the home. The police and *kebele* administrator asked one of the woman’s children where his mother was, and the child said his mother was in the home. Finally, they convinced the husband to bring the woman back to the hospital, but not the woman. It became apparent that she was frightened that the doctors would force her to have an operation (Caesarean Section). She started labour at home and had antepartum haemorrhage. Finally, she was taken to Adwa Hospital where she had a normal delivery.”

Some women have experienced disrespectful care at the hospitals. HEW8 described how women preferred to go to the health centre because they perceive more respectful care exists there compared to the hospital. One of the health workers at the *woreda* health office stated:

“Previously there was a lot of problem because of the mistreatment of rural women at the hospital. We learnt about this problem through the WDGs and acted on the feedback. This problem has been solved by the *woreda* head—not to mistreat women from rural areas in the hospital. One midwife was dismissed because of this. Now we meet the medical director of the hospital every three months to discuss this.”

### Decision making

We found that husband’s decision making power ranked high on farming related issues. Some husbands still decide on the number of children their wives should have and the place where the child should be born. Other husbands are learning about family planning and the benefits of ANC and institutional delivery. Although all maternal health services are provided free of charge, some husbands resist paying extra costs such as fares to return home after health facility delivery and the cost of food at health facilities. For instance, HEW9 pointed out that:

“If the woman doesn’t want to go to the health centre because of her children and needing to prepare food and so on, the husband may ask the HEW to convince her to go. But there are other husbands who don’t allow their wives to go—one husband insisted his wife should stay at home two nights when she was in labour.”

One of the key messages during WDG and other meetings at the *kebele* level is that no woman should die while giving birth. HEW2 stated:

“Some men are starting to show a supporting role when their wives are pregnant by asking us to talk to their wives about ANC and bringing them for labour to the health centre. If they know the Expected Delivery Date they wait for that time.”

HEW1 explained that:

“Men give good support, most husbands handle house affairs if the wife goes to ANC, men have to call the ambulance or invite people to help carry their wife to the road on a stretcher. Five years ago, some women took family planning in secret, now some women are even using IUCD.”

### Factors leading to skilled birth attendance

We found that the facilitators to SBA included HEWs and WDGs who identified pregnant women and referred them to health posts for their first ANC visit and then subsequent visits to health centres. The WDGs assist HEWs by advocating the benefits of SBA to women and notifying the HEWs if a woman’s labour starts at home. HEWs organize for women to travel to health centres for delivery either before their EDD or if labour starts at home. Husbands play a crucial role in helping their wives attend ANC and SBA.

### Women’s Development Groups

WDG leaders and HEWs provide information about ANC and the need to give birth at health facilities. HEWs use stories during WDGs and other meetings at the *kebele* level to enable people to compare the past and the present. During the workshop and interviews, HEWs related some of these stories. For example, HEW15 first started working as a HEW around 10 years ago:

“Back then, there was a lot of confusion about what to do and how to teach pregnant women. Almost no one attended ANC, no one had a health facility delivery and there was no road. There was so much resistance at the start and everyone said, ‘Why should we go to a health facility? Everyone was born at home so there’s no need to go. It’s an additional expense.’ Since then, there have been many changes, sometimes it’s hard to keep up with what is expected of me. I’ve gained a lot in confidence if I compare the past to the present I can see changes in myself as well—overtime people have come to accept and trust me. There have been small changes that have made a big difference for women. For example, if a woman is very fearful about going to the health centre, I can go with her and stay with her but that is getting harder to do, as I’m just too busy and there are midwives in the ambulance now. But I talked to the head of the health centre about allowing some traditional practices such as letting women put perfume on to burning incense *(etan)* during the labour and this has made a difference along with the picture of *Mariam* on the wall of the labour ward.”

HEW9 related a story that the other HEWs decided to adopt to share with women in their *kebeles*:

“Four years ago, before the ambulance service was introduced I was on my way to training in Mekelle when someone came and told me about a woman who was bleeding after home delivery and wanted my help. It was clear the others considered the situation as if the woman had already died. I tried to tell the family to take the woman to the hospital but they asked, ‘Why should we carry a dead woman?’ I had to insist that the woman be carried on a stretcher to the road but the rest of the family didn’t want to pay the extra costs of going to the hospital ‘if the woman was already dead.’ I asked some people to prepare a stretcher, and raised the mother’s legs up to increase the blood flow to the vital organs. People carried the woman but they wanted the baby to stay at home but I insisted they bring the baby. The woman was vomiting at her home and on the stretcher the whole way. Coincidentally at the time this was happening, people from the TRHB were visiting to conduct supervision so there was a car in the *kebele*. I asked them to take the woman to the hospital urgently—if the woman had stayed another five minutes she would have died. After this incident, the community changed what they thought about me. Now the woman is raising her children and she visits me regularly and praises me for saving her life and her baby’s life.”

### Ambulance services

The introduction of the ambulance service has created a new role for HEWs as they are now responsible for calling for ambulances to transport women either before their EDD or during onset of labour at home. Depending on the situation, a child or the woman’s husband will call the HEW in person or by telephone. If the husband is around, he may need to organize a group of men to carry the woman on a stretcher to the main road. Health centres are expected to allow women from far *kebeles* to stay for one or two weeks sometimes even a month prior to their EDD. The family is expected to provide food pre-partum and the health centres provide porridge *(gunfo)* and a coffee ceremony post-partum. At the same time, disincentives have been put in place to make husbands responsible if they disallow their wives to go to the health centre for delivery. HEW12 stated that:

“The Women’s Development Group helps to identify pregnant women and the HEW gives them a referral card to Adwa Hospital or to the health centre. All the women should go for ANC but some don’t go for all the visits. If women are from a distant place, they come and stay here at the health post but if the labour starts at home, they call the HEWs who are responsible for calling the ambulance. It comes in around 30 minutes. Two women gave birth on their way to the health post. If action is not taken to call immediately, the one at home will be held accountable.”

Similarly, HEW9 explained that:

“From the EDD we bring women to the health centre before labour starts. But if labour starts at home, they have our mobile number and call us and we call the ambulance to bring them to the health centre. At the health centre, one woman is waiting now, and one went to Adwa Hospital. If they are waiting at the health centre, someone is supposed to bring them food from home. After birth, the health centre will provide *gunfo* and coffee. The *gunfo* is prepared during third stage so it is ready to be eaten immediately, then the coffee after that.”

Sometimes the ambulance did not always come in time—partly caused by one ambulance being involved in an accident so being unavailable for many months. HEW1 stated that:

“The ambulance is not always available to come—it may be in one of the other *kebeles*—so we call the *woreda* health office and they arrange for another car to come to the *kebele*—any other driver in the *woreda* (such as a driver from the agriculture or education sectors) then the *woreda* health office will pay for the fuel of the other car. There are no problems for the mothers—but there is only one ambulance for 18 *kebeles*. The ambulance stays in Adwa, sometimes there is a fuel shortage but not to the extent of letting a mother have her baby at home. If a woman needs a Caesarean Section, she will be taken to Adwa Hospital, the same for assisted delivery in Adwa.”

HEW13 recounted how she called the ambulance for a woman who started labour at home. She waited on the roadside with the labouring woman but the ambulance had gone somewhere else so the woman delivered on the roadside. The family were very angry with the HEW for this—they said she was doing a favour to some families and not to others:

“If she can’t call it on time—why didn’t I have my labour at my home—it is not acceptable to have a baby on the roadside.”

## Discussion

Our findings showed that while there have been substantial efforts to improve maternal health and reduce maternal mortality in Adwa Woreda in Tigray Region, the findings are consistent with those from other studies that identify reasons why women in Ethiopia prefer home delivery [25, 27, 28, 36], and why women do not seek skilled assistance during complicated birth [37, 38]. Until recently, most women gave birth at home assisted by a TBA or family member. Women who previously delivered at home without any problems and younger women living with their mother or mother-in-law may be pressured to stay at home to give birth.

In the 2011 EDHS, 13% of currently married women make their own decisions about their own health care while 25% said that their husbands mainly take such decisions [3]. We found that women told HEWs that many husbands are starting to show a supporting role when their wives are pregnant and some men approach HEWs directly to ask for assistance. A study from Tigray was consistent with our findings where husbands were in favour of institutional delivery [27]. However, the absence of men during many months of the year does not motivate some women to attend ANC or stay at health centres before their child is born if no one is at home to look after other children. At times, it can be difficult to find enough men to prepare a stretcher and carry an expectant mother to the main road so some communities have had to appoint older school boys to do this.

Other studies emphasize how women’s groups can lead to substantial reductions in maternal and newborn mortality in rural, remote and resource-limited settings [39–41]. One study in Ethiopia argued that the HDA is likely to be an effective strategy for improving maternal and newborn health practices as it involved strategies to mobilize communities, to encourage pregnant mothers to give birth in health facilities; creating effective supportive and referral linkages within the primary health care units; staffing health centres with midwives to ensure continuous availability of basic emergency obstetric care services, and the provision of ambulances to *woredas* to mitigate transportation barriers [17].

Women and HEWs stated that the introduction of WDGs in Adwa Woreda assists HEWs by encouraging women to avoid giving birth at home and instead to go to health facilities. Many HEWs described how confused they were in the past—and that they did not know how to encourage women to come to the health post or health centre for ANC or delivery. Now HEWs keep a chart on the wall of the health post that shows a pregnant woman’s name, last menstrual period, EDD, name of her husband, name of WDG leader, name of religious father, name of *kebele* cabinet member responsible for the pregnant mother, and name of the individual who will be responsible during when labour starts. WDG leaders now meet weekly or fortnightly with HEWs.

According to the *Road Map*, ambulances should link health posts in rural *kebeles* with health centres (at the *woreda* level), and health centres with hospitals (at the zonal and regional level) through a referral system so that women can be transferred if they need EmONC [6]. This referral system is considered to be the key to reducing delays that currently contribute to maternal and neonatal mortality and morbidity [42, 43]. We found that there was strong commitment from Adwa Woreda Health Office to ensure the availability of ambulances at all times and in all localities. There were times when HEWs stated that the ambulance was delayed but this appeared to be because it was in another part of the *woreda*.

Some health centres have put aside space for mothers to wait in the final week of pregnancy. HEWs and health centre staff have identified a number of incentives that have improved women’s likelihood of delivering in a health centre. Based on the health worker’s perceptions, these incentives might be considered as trivial, but they appear to be cost-effective enticements. They include allowing women to wear perfume and to burn *etan* during labour, putting pictures of Mariam on the wall of the labour ward, providing *gunfo* for the woman to eat straight after delivery, providing a celebratory coffee ceremony, and providing clothes for the newborn baby.

Women, HEWs and other health workers identified the need for skilled birth attendants to be respectful because many women are fearful of giving birth in a health facility—and especially fearful of being referred to the hospital for a Caesarean Section. Some women identified abusive care as a disincentive to attend health facilities. The Adwa Health Office has scheduled regular meetings to ensure that skilled birth attendants treat women from rural areas with respect. Other studies have also found that disrespectful care can be a deterrent to skilled birth attendance as women prefer a health provider that shows respect for their patients [44–46].

A combination of political commitment and resources to health facilities along with the WDGs has seen an increased number of women giving birth in health facilities in Adwa Woreda. WDGs are mobilizing communities to ensure early referral of women to health centres for delivery—thus reducing the first delay. To reduce the second delay, especially the shortage of transportation in rural areas, the introduction of ambulances to each *woreda* reflects the commitment and multi-sectoral response to improving maternal health care. The TRHB is currently working with health centres and hospitals on a maternal death review to determine why women die in childbirth [30, 46]. The combination of these factors could help ensure that the motto “No woman should die while giving life” becomes a reality for all women in rural Adwa Woreda and in Tigray Region.

### Study limitations

There were limitations in our study as our data and conclusions are based on a small number of HEWs and women’s responses from one *woreda* in Tigray Region. Although HEWs are under considerable pressure to refer all women to health centres for ANC and delivery we occasionally felt that their responses were what they thought ‘we should hear.’ We spent time during field visits with the HEWs reiterating the benefits of the research and that all responses would be deidentified. There was no direct relationship between HEW performance evaluation and our study objectives. As acknowledged by Price and Hawkins [33], criticism surrounding the validation of data is the point where the method departs from positivism. This is identified as a strength of the method which allows analysis of contradiction and difference in people’s discourses ‘within a social network rather than on gathering ‘social facts.” Qualitative researchers aim to ‘place the interpretative process at the centre of their practice. The interpretative process refers to the way that people interpret and give meaning to events and things’ [47]. We selected HEWs to be data collectors because they are commonly recruited from their own *kebele* and were more likely to be culturally acceptable because they deliver health packages that relate to issues affecting women and children. For issues that influence rural women in Ethiopia’s access to health services, fertility and mortality rates, access to sanitation and water, rates of illiteracy and women’s subordination we assume a fair level of generalizability across Ethiopia [17, 18, 48–51].

## Conclusion

In Adwa Woreda, the key barriers for women not to attend ANC and institutional delivery were absence of husbands for many months in the year, distance, and lack of transportation due to geographical factors. The cultural tradition of *zwar* was also perceived as a barrier for some women to attend ANC services. Disrespect and mistreatment by health workers and health facilities were also additional barriers for women among other challenges. However, the active engagement of WDGs to mobilize pregnant women to attend health facilities and the commitment of HEWs for facilitating referral linkage were considered as motivating factors as was the role of husbands who supported their wives to attend health facilities. The joint efforts of HEWs and the WDGs has contributed to changing the attitudes of women from the embedded traditions of giving birth at home, to seeking modern health care services.

Despite the political commitment and mobilization of WDGs to work with HEWs to improve rates of ANC and institutional delivery, there is still a long way to go to tackle the existing challenges including mechanisms to overcome *zwar*. A forum is needed to improve the role of husbands and ways to increase women’s empowerment. Health workers should be trained to approach women with empathy, dignity and respect. These findings have broad implications for other areas in rural Ethiopia and other sub-Saharan African countries where HEWs or Community Health Workers facilitate skilled birth attendance by referring women from rural locations to mid-level health facilities.
